# Optical and Acoustic Sensor-Based 3D Ball Motion Estimation for Ball Sport Simulators †

**DOI:** 10.3390/s18051323

**Published:** 2018-04-25

**Authors:** Sang-Woo Seo, Myunggyu Kim, Yejin Kim

**Affiliations:** 1 Creative Content Research Division, Electronics and Telecommunications Research Institute, 218 Gajeong-ro, Yuseong-gu, Daejeon 34129, Korea; mgkim@etri.re.kr; 2 School of Games, Hongik University, 2639 Sejong-ro, Jochiwon-eup, Sejong 30016, Korea; yejkim@hongik.ac.kr

**Keywords:** 3D ball motion, infrared scanning, sports simulator, acoustic sensor, beamforming, sound source localization

## Abstract

Estimation of the motion of ball-shaped objects is essential for the operation of ball sport simulators. In this paper, we propose an estimation system for 3D ball motion, including speed and angle of projection, by using acoustic vector and infrared (IR) scanning sensors. Our system is comprised of three steps to estimate a ball motion: sound-based ball firing detection, sound source localization, and IR scanning for motion analysis. First, an impulsive sound classification based on the mel-frequency cepstrum and feed-forward neural network is introduced to detect the ball launch sound. An impulsive sound source localization using a 2D microelectromechanical system (MEMS) microphones and delay-and-sum beamforming is presented to estimate the firing position. The time and position of a ball in 3D space is determined from a high-speed infrared scanning method. Our experimental results demonstrate that the estimation of ball motion based on sound allows a wider activity area than similar camera-based methods. Thus, it can be practically applied to various simulations in sports such as soccer and baseball.

## 1. Introduction

The accurate estimation of ball motion including velocity, angle of projection, and spin is essential for the ball simulation in virtual sports. The recent success of a screen golf system [1] has derived the development of other simulations in sports such as baseball and soccer [2,3]. Most of the current sport simulations rely on the computer vision-based techniques [4,5], which adopt multiple ultrahigh-speed cameras fixed on the high locations. The performance of the image-based estimation is greatly influenced by the capabilities of cameras in capturing area, angle of view, and sensitivity of illumination conditions. For example, the golf simulator is generally operated under an indoor environment to detect a ball motion from a constrained hitting area. Unlike the golf simulator, where the ball trajectory can be delimited to a narrow and specific area, other multi-player simulators (i.e., baseball and soccer) require a wider area for striking a ball, which makes the ball motion analysis in 3D space more difficult.

In this paper, we present an estimation system for 3D ball motion based on infrared (IR) and acoustic sensors. The proposed system is comprised of three steps to estimate 3D ball motion: sound-based ball firing detection, sound source localization, and IR scanning for 3D motion analysis. First, to recognize impulsive sound caused by firing a ball, we determine the mel-frequency cepstrum coefficients (MFCCs) and use them as input to a feed-forward neural network (FFNN). Once the ball firing sound is detected, a 2D microelectromechanical system (MEMS) microphones and a delay-and-sum beamforming method is used to localize a source of impulsive sound. For this, a parametric method is used to determine the sound time and position. Given the location of ball, a high-speed IR scanner is utilized to determine the ball trajectory in 3D space. As shown in our experimental results, the proposed system demonstrates that the sound-based estimation of 3D ball motion provides a wider activity area than similar camera-based methods while keeping its high accuracy. Thus, it can be practically applied to various multi-player simulations in sports such as baseball and soccer.

The classification of sound detections can be performed using different types of features such as pitch range, the time difference of arrival (TDOA), spectrogram, linear frequency cepstral coefficients, gammatone frequency cepstral coefficients, and MFCC. Kim et al. utilized three features (pitch range, TDOA, and spectrogram) to increase the classification accuracy [6]. However, the large size of the features causes the degradation of the performance. Zhao et al. showed that MFCCs provide a feature vector with few elements with superior noise robustness [7]. They reduced the execution time of the FFNN but compromised accuracy compared to the image-based approach, which transforms the sound data into an artificial neural network to recognize human sound [8]. In our system, MFCC is adopted to provide a real time performance by reducing the size of input vectors of the neural network.

Based on the type of mapping function, sound source localization methods can be divided into two types: parametric [9,10] and non-parametric [11]. Acoustic holography [12], one of typical non-parametric methods, can detect not only the position of the sound source but also the characteristics of the sound field. However, it requires a long calculation time and many microphones for the source localization. On the other hand, the parametric method can locate the origin of the sound source faster than the acoustic holography, by using a parameter of the signal with a relatively small number of sensors. Some of the previous approaches presented a parameter-based 3D sound source localization method to estimate the direction and distance in frequency domain [13,14,15]. However, it is difficult to distinguish the impulsive sound source such as gun firing and ball kicking sound from the background noise when its frequency is high due to the lack of a frequency feature for it. Seo et al. presented a beamforming technique for the impulsive sound source localization [16]. In their system, the spherical wavefront model is used over the planar one to analyze the sound wave, as the latter is more suitable for position estimation [17]. Heilmann et al. proposed a method of 3D sound source location with a large number of expensive microphones arranged in 3D space for the real time performance [18]. In their system, the microphones should be placed precisely in a spherical arrangement. Our system has adopted a similar parametric method due to the real time performance required for the sports simulator. However, our time-based beamforming technique relies on the time delay such as TDOA with the spherical wavefront model to analyze the sound wave.

To determine the time and position of a fast-moving ball in 3D space, a laser scanner is adopted by a baseball simulator such as the Real Yagu Zone system [19]. However, the employed laser devices have a comparatively lower lifetime than IR scanners, given the high junction temperature of the laser. In our system, an inexpensive high-speed infrared (IR) scanner is adopted to estimate the position and timing of the ball [20].

Our system makes two main contributions. First, comparing to the camera-based simulators, our system is less sensitive to the environmental changes such as lighting and background noises by utilizing the sound-based sensors. This provides more accurate estimations of ball motion in 3D space, as shown in our experimental results. Next, our system allows a user to hit a ball without a placement restriction like the previous system [4]. This increases the applicability of our system to more diverse sport simulations such as soccer and baseball.

The remainder of this paper is organized as follows. An overview of the proposed system is introduced in Section 2. In Section 3, we describe the optical and acoustic sensor-based 3D ball motion estimation for ball sport simulators. The experimental results are demonstrated in Section 4. We conclude the paper with a discussion of potential improvements in Section 5.

## 2. System Overview

**Figure 1:**
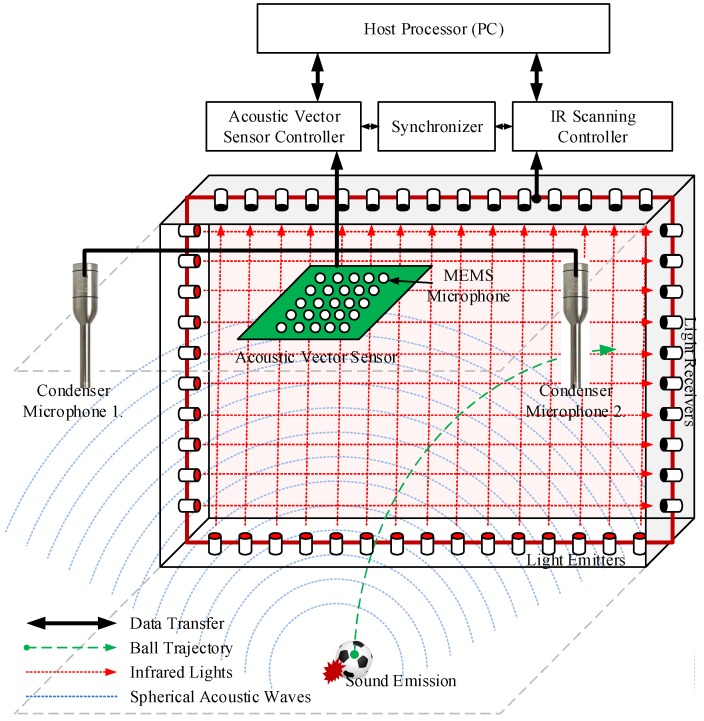
Overview of the proposed system: the spherical sound localization using the acoustic.

**Figure 2:**
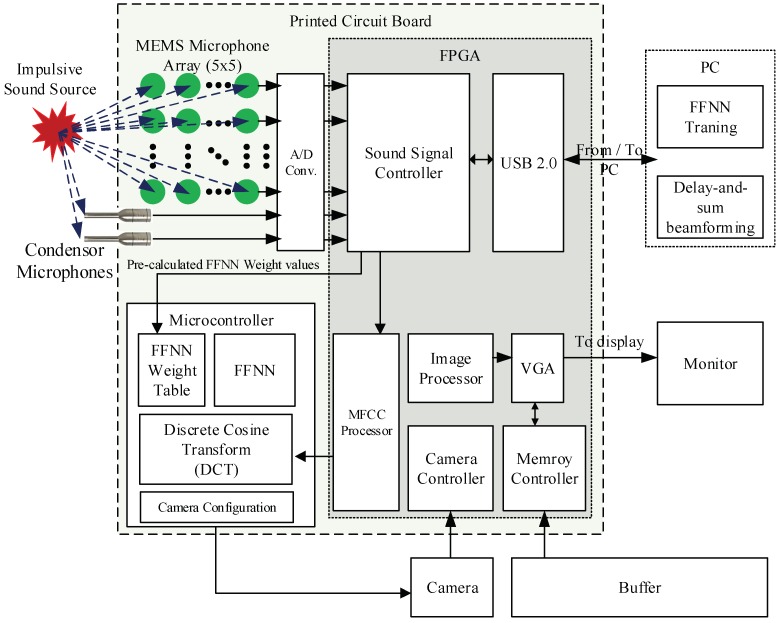
The hardware design for impulsive sound detection using a MFCC-FFNN scheme.

Figure 1 shows an overview of the proposed system to estimate 3D ball motion. Prior to the estimation process, the acoustic sensors should be calibrated, and the two controllers (i.e., acoustic vector sensor and IR scanning controllers) should be synchronized. The proposed system is comprised of three steps: sound-based ball firing detection, sound source localization, and IR scanning for motion analysis. Figure 2 shows the proposed estimation architecture for 3D ball motion. When a ball is fired, the acoustic vector sensor receives impulsive sound signals, which is emitted whenever the ball is hit or kicked. The ball hit is detected when a predefined threshold is reached. The acoustic vector sensor controller recognizes whether the received sound is a ball firing through an MFCC-FFNN algorithm. The IR scanner detects a ball-shaped object and determines its position on the virtual plane (i.e., the red plane in Figure 1) when it passes through the designed IR scanning frame. Both controllers transfer their data containing acoustic and IR light signals to the host processor (i.e., a PC in the proposed system). Subsequently, the host processor estimates the initial ball position using impulsive sound source localization method and the ball speed and angle of projection from the output results of both the sound source localization and the IR scanner.

## 3. D Ball Motion Estimation

### 3.1. Sound-Based Ball Firing Detection

**Figure 3:**
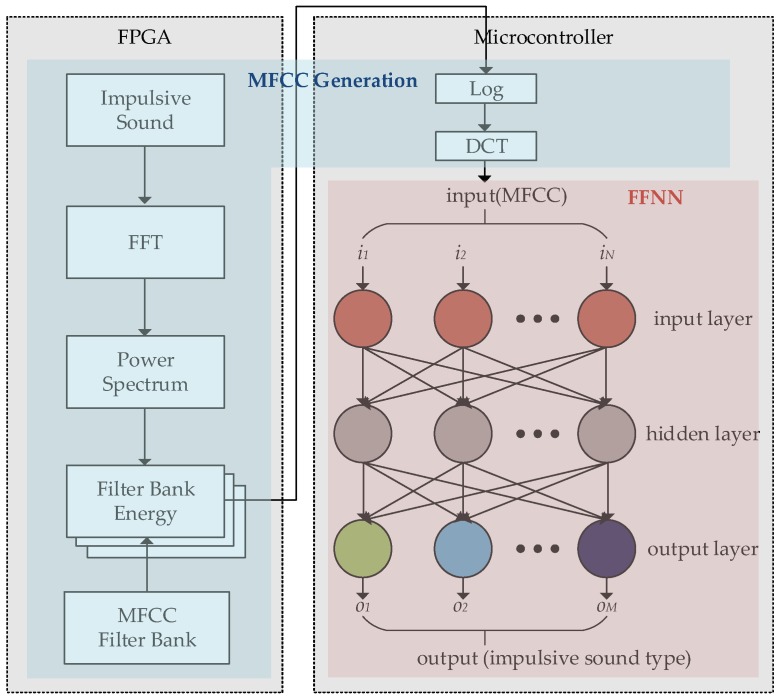
The MFCC-FFNN scheme using MFC (implemented by FPGA) as an input to FFNN (implemented by microcontroller).

Figure 3 shows a diagram of the MFCC-FFNN algorithm for the proposed system, where the MFCCs represent the input feature vector of the FFNN. Inspired by the neural network approach [21], our system detects and identifies whether an impulsive sound corresponds to ball firing from a learning-based model. Given that the FFNN training would take a long time on the embedded microcontroller, we executed this training on the host processor and transferred the weights and training outputs to the embedded system. After analyzing the MFCC and FFNN, we found that the MFCC estimation takes much longer than the FFNN execution given the large amount of iterations to obtain the Fourier transform and to sum the filter bank energy. Considering the MFCC and FFNN processes, we implemented most of the MFCC estimation using a field-programmable gate array (FPGA) to improve computational efficiency. Only the logarithmic and discrete cosine transform stages of the MFCC estimation are conducted at the microcontroller to provide the FFNN inputs. The parameters of the implemented MFCC-FFNN algorithm are listed in Table 1.

To achieve high recognition accuracy, the impulsive sound is received at 192 kHz sampling frequency with 24-bit precision. In addition, since the duration time of the sound is very short (less than one second [22,23]), 20 frames with the size of 1024 are overlapped by 75%, which improves the time resolution.

### 3.2. Sound Source Localization

#### 3.2.1. Estimation of 2D Sound Source Position

**Figure 4:**
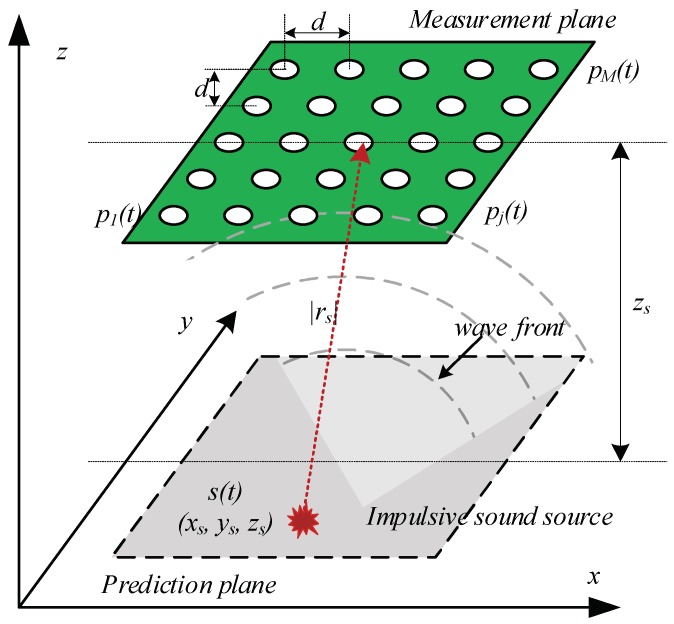
Sound measurement setup and spherical acoustic waves emitted by a monopole source.

Figure 4 shows the wave propagation of an impulsive sound source considering the spherical wavefront model. We adopted this model to estimate the position when a ball is fired using a 2D MEMS microphone array and delay-and-sum beamforming, similar to a FPGA-based real-time acoustic camera prototype [24]. The measured signal at time *t* for each microphone in a free field can be defined as

(1) $$p_{j}\left(t\right)=\frac{1}{\left|r_{s}\right|}s\left(t-\frac{\left|r_{s}\right|}{c}\right),$$

where *s(t)* is the signal at the position of the impulsive sound source, |*r_s_*| is the location vector between microphone *j* and the source, and *c* is the propagation speed of the acoustic wave. The delay-and-sum beamforming output with respect to candidate position *P_s_* at sample *i* is given by

(2) $$bf\left(P_{s},i\right)=\frac{1}{M}\sum_{j=1}^{M}p_{j}\left[i-\delta_{j}\left(P_{s}\right)\right],$$

where *p_j_*[*i*] is the measured sound stream of microphone *j* at index *i* from the measured microphone stream, *M* is the number of microphones, and $\delta_{j}\left(P_{s}\right)$ is the sound propagation delay between *P_s_* and *P_mj_*, which is the position of microphone *j*, at sampling frequency *f_s_* defined by $\;\frac{f_{s}}{c}\left|P_{s}-P_{mj}\right|$. The output intensity of delay-and-sum beamforming for all the candidate positions of an impulsive sound source can be averaged for *L* samples, where the number can affect the final result of both the sound source detection and localization. 

The number of signals regarded as impulsive sounds can be determined by

(3) $$L=i_{e}-i_{s},\;\{\begin{array}{c} i_{s}:=S-O,\;\sqrt{\sum_{i=1}^{S}P{\left(i\right)}^{2}}>K\times B_{RMS}\\ i_{e}\;:=E+O,\;\sqrt{\sum_{i=1}^{S}P{\left(i\right)}^{2}}<K\times B_{RMS} \end{array},$$

where *S* is the position of the sample detected by the over-threshold (K × B_RMS_), *E* is the position detected by under-threshold, *i_s_* and *i_e_* are the indexes of the initial and final samples, respectively, *O* is the offset number of samples, *P(i)* is the summation of all the measured microphone signals at sample *i*, *K* is a predefined threshold, and *B*_RMS_ is the root-mean-square value of background noise. In the proposed system, *O* and *K* are defined as 128 and 3, respectively, based on a heuristic method to extract the valid samples for the impulsive sound source. Finally, the position of the fired ball can be estimated as

(4) $$P_{bf}=\underset{P_{s}}{\max}\left[bf\left(P_{s},i\right)\right]$$

#### 3.2.2. Estimation of Prediction Plane Depth and 3D Localization

**Figure 5:**
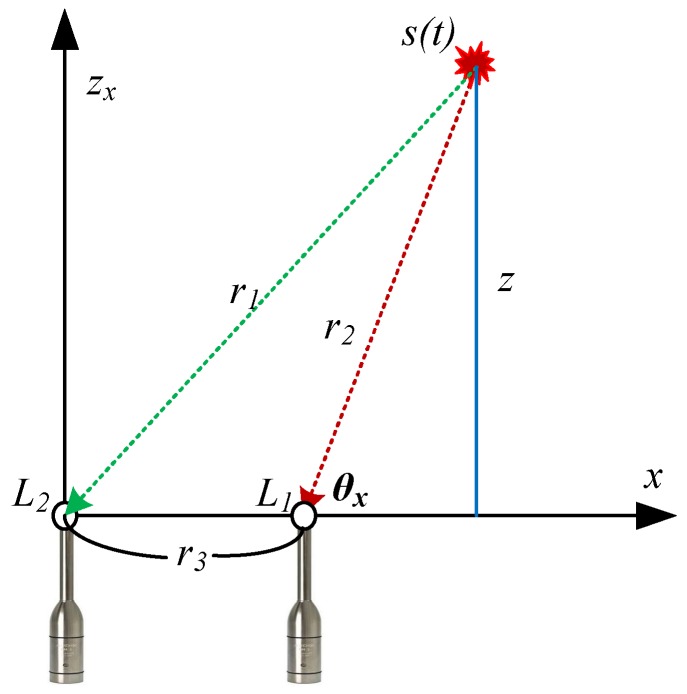
Estimation of the prediction plane *z*-axis value.

Figure 5 shows the estimation of the *z*-axis value (i.e., the distance between the measurement and the prediction planes depicted in Figure 4). Variables *L*_1_ and *L*_2_ represent the sound pressure levels of the two microphones for depth estimation, *r_3_* is the distance between the microphones, *θ_x_* is the sound source direction obtained in Section 4.1, and *r_1_* and *r_2_* are the distances between the sound source and microphones corresponding to *L*_1_ and *L*_2_, respectively. The relation among *r*_1_, *r*_2_, and *r*_3_ is defined according to the law of cosines as

(5) $$r_{1}{}^{2}=r_{2}{}^{2}+r_{3}{}^{2}+2r_{2}r_{3}cos(\theta_{x})$$

From Equation (5), we can estimate the distance between the prediction and measurement planes as

(6) $$z_{x}=r_{2}sin(\theta_{x}),r_{1}=g\cdot r_{2},$$

where *g* is given by

(7) $$\frac{r_{1}}{r_{2}}=g=10^{\frac{\left|L_{1}-L_{2}\right|}{20}}$$

Depth *z_x_* for the *x* axis can be rewritten by substituting Equations (5) and (7) into Equation (6):

(8) $$z_{x}=\frac{b\pm \sqrt{b^{2}+4\cdot r_{3}^{2}\cdot \left[g^{2}-1\right]}}{2\cdot \left[g^{2}-1\right]}\cdot sin\left(\theta_{x}\right),$$

where *b* = *2* *r*_3_ cos(*θ_x_*)*.* Finally, the depth can be obtained from *z_x_* and depth for *y* axis *z_y_* as

(9) $$Z=\sqrt{z_{x}^{2}+z_{y}^{2}}$$

### 3.3. IR Scanning for Motion Analysis

Our system exploited the previous IR scanner [20] for detecting the initial position and estimating the trajectory of a ball in 3D space. We added a synchronization unit to integrate impulsive sound source localization and estimated the velocity of a ball based on the time difference between the two systems as the IR scanning system only detects the position, not the velocity, when the ball passes through the scanning frames.

The velocity, elevation, and azimuth of a fired ball is estimated as follows: The ball angle of projection can be expressed as

(10) $$\{\begin{array}{c} \theta_{el}=tan^{-1}\left(\frac{P_{sz}-P_{lz}}{P_{sy}-P_{ly}}\right)\\ \theta_{az}=tan^{-1}\left(\frac{P_{sx}-P_{lx}}{P_{sy}-P_{ly}}\right) \end{array},$$

where *P_s_(x, y, z)* is the position determined from the IR scanning system and *P_l_(x, y, z)* is the estimated position of the impulsive sound source caused by the ball firing. In our implementation, *P_sy_* is constant because the IR scanning system is installed at a fixed frame.

The velocity of the ball along the *x*, *y*, and *z* axes can be calculated as

(11) $$\{\begin{array}{c} v_{x}=\frac{P_{sx}-P_{lx}}{t_{s}-t_{l}}\\ v_{y}=\frac{P_{sy}-P_{ly}}{t_{s}-t_{l}}\\ v_{z}=\frac{P_{sz}-P_{lz}}{t_{s}-t_{l}} \end{array},$$

where *t_s_* and *t_l_* represent the detection time of the IR scanning system and the measured time using the sound source localization of the ball being fired, respectively. Then, the 3D ball speed is given by

(12) $$v_{Ball}=\sqrt{v_{x}{}^{2}+v_{y}{}^{2}+v_{z}{}^{2}}$$

## 4. Experimentation

### 4.1. Experimental Setup

**Figure 6:**
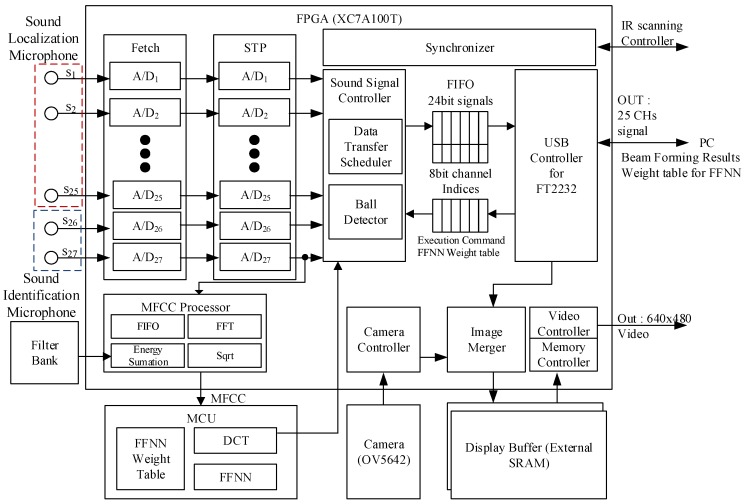
Overall system implementation for the estimation of 3D ball motion.

**Figure 7:**
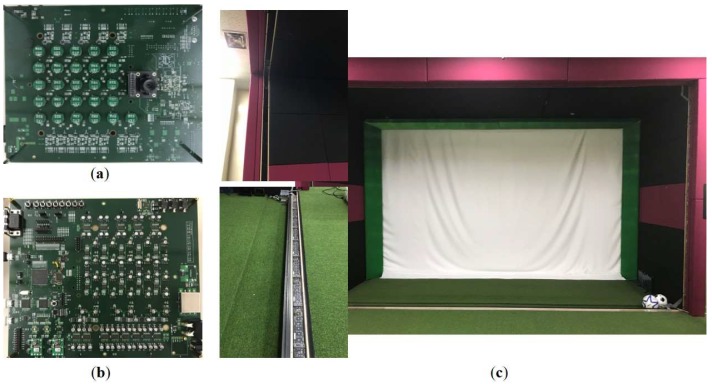
System implementation: (**a**) arrangement of the MEMS microphone array; (**b**) controller for impulsive sound source localization; and (**c**) ball motion scanning frame.

Figure 6 shows the overall system implementation with the connections and interactions between the components. Figure 7a shows the placement of the MEMS microphone array, where 25 MP33AB01 microphones (S_1_ to S_25_ in Figure 6, analog bottom type, STMicroelectronics, Geneva, Switzerland) are deployed in a 5 × 5 arrangement with distance *d* of 2 cm between adjacent microphones. The array gain is proportional to the number of sensors [25]. Therefore, it is advantageous to use many sensors to reduce the influence of noise and increase the output signal-to-noise ratio to estimate the position of the impulsive sound source. However, since the large size of microphones will increase the processing time of the proposed system, we assume that it is appropriate to use 25 sensors. In addition, we consider the distance appropriate because we designed the sound localization device based on the spherical wavefront model, which is suitable for the near field between the microphone arrays placed in the ceiling and the impulsive sound of the ball originated on the ground. The two AUDIX TM1 condenser microphones (S_26_ and S_27_ in Figure 6, Audix Microphones, Wilsonville, OR, USA) are used to classify the impulsive sound to recognize the ball sound and estimate the depth of the prediction plane, as described in Section 4.2. These microphones are situated at the sides (left and right) of the microphone array and far from the center of the microphone array, as illustrated in Figure 1, to achieve accurate results of triangulation. Since we adopted time-domain based beamforming techniques, the acoustic signals were sampled at 192 kHz with 24-bit resolution to achieve good performance [26]. In addition, we implemented an analog-to-digital converter in the FPGA board using the manufacturer’s development kit [27].

Figure 7b shows the implemented acoustic sensor controller, which receives acoustic signals from 27 microphones and synchronizes their phases. It recognizes the sounds produced by the ball and filters other types of sounds from a single motion-blurred image [28] and transfers the sound information to the host processor via a USB connection. The controller is mostly implemented on the cost-effective Artix-7 XC7A100T FPGA (Xilinx, Inc., San Jose, CA, USA) and STM32F microcontroller (STMicroelectronics, Geneva, Switzerland) to support the final stages of MFCC, the FFNN algorithm, and communications, as shown in Figure 3.

The width and height of the IR frame are 4 and 2.5 m, respectively, and it contains 176 pairs of IR sensors, with every emitter placed at 3 cm apart from each other. This high density of IR sensors aims to detect the motion of a baseball sized 7.23 cm. However, there is a tradeoff between accuracy and the scanning rate, as more sensors reduce the scanning rate. In our system, the scanning rate from the previous system [20] is improved to approximately 20 kHz, which provides a better processing rate than the ultrahigh-speed cameras do in the other sports simulators. In addition, our IR scanning system is mainly used to detect the ball location in 3D space while the previous one is used to detect the velocity of the flying ball when the ball passes the scanning frame.

### 4.2. Calibration

Calibration is essential to obtain accurate beamforming results, as we estimate the initial ball position from the impulsive sound caused by the ball firing based on the sound pressure level. To retrieve accurate sound pressure measurements, the voltage of the relative sensitivity and phase of all microphones must be calibrated. Despite being accurately calibrated using specialized equipment, such as the Type 4231 sound calibrator (Brüel and Kjær Sound and Vibration Measurement A/S, Nærum, Denmark), MEMS microphones accumulate dust over time, and their properties can be altered, thus requiring periodic recalibrations. Moreover, to avoid dismounting and mounting the microphone system for calibration, we adopted the free-field method that uses the spherical characteristic of sound propagation [29]. This method allows us to calibrate all the microphones without taking them out from their printed circuit board by using a single Type 4295 omnidirectional loudspeaker (OmniSource™; Brüel and Kjær Sound and Vibration Measurement A/S, Nærum, Denmark). After calibration, the proposed system generates a hardware-based trigger signal to the acoustic vector sensor and IR scanning controllers. When the trigger is activated, the timers of both controllers are reset to zero, such that the asynchronous controllers share synchronized timing, which is crucial for accurately estimating the ball speed.

### 4.3. 3D Ball Motion Estimation

**Figure 8:**
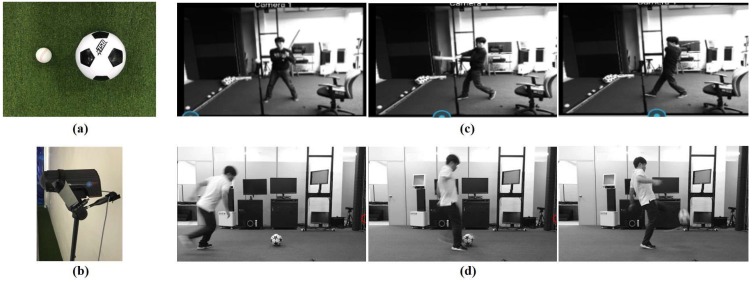
Speed estimations of balls: (**a**) a baseball (7.23 cm) and a soccer ball (22 cm); (**b**) Stalker Pro II sports radar gun as reference; (**c**) a sequence of baseball swings; and (**d**) a sequence of soccer ball kicks.

Figure 8 shows the simulation setup used for the estimations of ball motion in 3D space by using two different sized balls, a baseball (7.23 cm) and a soccer ball (22 cm). Several users (i.e., 10 amateur players) are hired to perform various swings and kicks in an indoor environment. Any impulsive sound not from a ball is subdued except background white noises. Each player is given 20 tries to swing or to hit a ball, producing a ball impulsive sound. 

**Figure 9:**
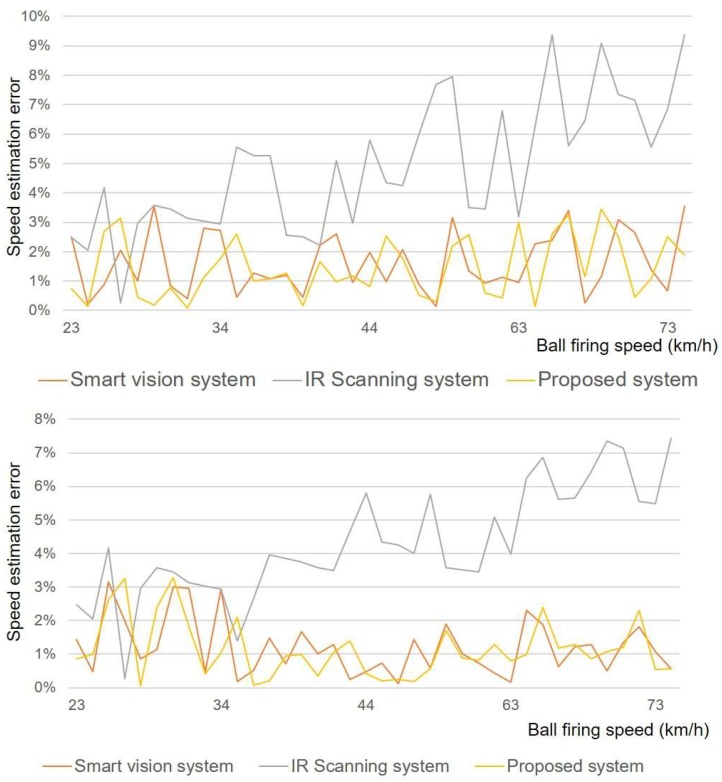
Speed error comparisons of baseball (**top**) and soccer ball (**bottom**) between smart vision system, standalone IR scanning system, and proposed system using Stalker Pro II sports radar gun as reference.

Figure 9 shows the performance of the proposed ball motion estimation system by comparing the measured speed of ball motion that is obtained from the previous IR scanning system [19] and the camera-based smart vision system [4], respectively. The error was determined from the mean speed of numerous swings and kicks that is measured with a commercial radar gun (the Stalker Pro II sports radar gun) [30]. Overall, the error of the proposed system remains below 4% over the entire measurement range and is comparable to the error of the smart vision system, whereas the error when using only the IR scanning system increases with the ball speed given its limited scanning rate. 

## 5. Conclusions

This paper presents an estimation system of 3D ball motion by using acoustic and IR sensors. The proposed system demonstrates the sound-based ball firing detection and localization to determine the initial position of a ball and a high-speed IR scanning method to detect the position of the ball when it passes through the scanning frames. In our system, the acoustic vector sensor controller classifies the ball firing sound for impulsive sound recognition based on FFNN algorithm with MFCC as the input feature vector. It estimates the position and timing of a ball using time-domain beamforming method, which is based on the spherical wavefront model. Once the ball is located, the high-speed IR scanning controller estimates ball positions and timings of the ball whenever it passes through the scanner. As the experimental results show, the accuracy of the proposed system is above 95%, similar to that of the camera-based smart vision system. The proposed system meets with the requirements of most screen-based ball sports simulators. 

One of the ongoing improvements in our system is adding an extra IR scanner to obtain additional position information. This way, the ball trajectory could be estimated in a greater accuracy using a physics engine. Currently, the beamforming process is estimated by CPU, which can be implemented into FPGA for a real time recognition of ball motion.

## Figures and Tables

**Table 1:** Parameters of the MFCC-FFNN algorithm.

Method	Parameter	Value
MFCC	Sampling Rate	192 kHz
	Number of samples per frame	1024
	Frame overlapping	75%
	Number of filter banks	20
	Number of frames per feature sound	20 (30.7 ms)
FFNN	Activation function	$\{\begin{array}{c} 0,\;\;\;\;\;\;x<-45,\;\;\;\\ 1,\;\;\;\;\;\;x>45,\;\;\;\;\\ \frac{1}{1+\exp\left(-x\right)},\;\mathrm{otherwise} \end{array}$
	Number of input neurons	400
	Number of output neurons	10
	Number of hidden layers	1
	Number of hidden neurons	500
